# A comprehensive preimplantation genetic testing approach for SEA-type α-thalassemia by fluorescent gap-polymerase chain reaction combined with haplotype analysis

**DOI:** 10.3389/fgene.2023.1248358

**Published:** 2023-11-23

**Authors:** Jing Wang, Yuanlin Ma, Jing Guo, Rong Li, Canquan Zhou, Yanwen Xu

**Affiliations:** Guangdong Provincial Key Laboratory of Reproductive Medicine, The First Affiliated Hospital, Sun Yat-Sen University, Guangzhou, China

**Keywords:** SEA-type α-thalassemia, preimplantation genetic testing, Gap - polymerase chain reaction (PCR), haplotype analysis, single nucleotide polymorphism (SNP) array

## Abstract

**Introduction:** This study aimed to evaluate the feasibility and necessity of using fluorescence Gap-polymerase chain reaction combined with haplotype analysis in preimplantation genetic testing for SEA-type α-thalassemia.

**Methods:** A total of 26 preimplantation genetic testing biopsy cycles were performed in 25 families from June 2021 to February 2022. All couples were carriers of SEA-type α-thalassemia. Fluorescent Gap-polymerase chain reaction was used for detecting fragment deletion. Subsequently, according to the results of polymerase chain reaction, reference embryos were identified to establish haplotype using single nucleotide polymorphism array, and aneuploidy was screened simultaneously. In cases wherein the polymerase chain reaction results were inconsistent with the haplotype results, the reasons were investigated, either by retest of the biopsied samples or rebiopsy of the embryo.

**Results:** Among the 172 embryos, 162 had consistent results when tested using both methods, resulting in a consistency rate of 94.2%. Conversely, 10 embryos had inconsistent results, mainly due to chromosome 16 aneuploidy (*n* = 7), allele dropout in Gap-polymerase chain reaction (*n* = 2), or incorrect haplotype due to poor sample amplification quality (*n* = 1). The clinical pregnancy rate of each frozen-thawed embryo transfer was 57.7% (15/26). Six families underwent prenatal diagnosis, which confirmed the results of preimplantation genetic testing.

**Conclusion:** Fluorescent Gap-polymerase chain reaction combined with haplotype analysis is feasible and necessary for SEA-type α-thalassemia preimplantation genetic testing.

## Introduction

α-Thalassemia is a common inherited autosomal recessive disorder caused by partial or complete inhibition of α-globin peptide chain, resulting in insufficient amount of hemoglobin (Frédéric and David, 2014; Sachith and Douglas, 2018). It is characterized by microcytic, hypochromic anemia, with a clinical phenotype varying from asymptomatic to lethal hemolytic anemia (Frédéric and David, 2014). It has been estimated that about 5% of the worldwide population carry an α-thalassemia variant. α-Thalassemia is known to highly affect tropical and subtropical regions, such as southern China, Southeast Asia, and Mediterranean countries (David, 2005; Bernadette and Matthew, 2008; Frédéric and David, 2014). The pathogenic variants of α-thalassemia are divided into the deletion and nondeletion types, with the former accounting for the majority of the variants. The most common types of α-thalassemia in southern China include the SEA-, 3.7-, and 4.2-type deletions. Among them, the SEA-type deletion has the highest prevalence (Shang et al., 2020). When both parents are carriers, the fetus has a 1/4 chance of developing homozygous SEA-type thalassemia (Hb Bart’s hydrops fetalis). The fetus usually dies between 23 and 28 weeks of gestation. Furthermore, the presence of Hb Bart’s hydrops fetalis can lead to serious maternal complications (Deng et al., 2006; Shang et al., 2020). Therefore, it is necessary to reduce the birth rate of edematous fetuses in couples carrying SEA-type α-thalassemias.

Preimplantation genetic testing (PGT), which is used to select unaffected embryos via genetic testing on biopsy cells from preimplantation embryos, is aimed at preventing the transmission of genetic disorders, thereby allowing couples carrying genetic diseases to have a healthy offspring. Conventional methods of PGT for SEA-type α-thalassemias include Gap-polymerase chain reaction (PCR), real-time PCR, and digital PCR (Deng et al., 2006; Wirawit et al., 2012; Ta-n et al., 2017). However, allele dropout (ADO) was inevitable in all these methods, thus leading to potential misdiagnosis with direct PCR detection. Linkage analysis of polymorphic markers near genes was employed to identify ADO and avoid misdiagnosis (Deng et al., 2006; Wirawit et al., 2012). However, in the above two reports, only one to two polymorphic markers were used for analysis. Aspasia et al. (2012) conducted a study using short tandem repeat (STR) polymorphic markers near the α-globin gene cluster for linkage analysis. An attempt to establish a general protocol for PGT detection of a-thalassemia was made. However, the number of STR markers was limited with uneven distribution throughout genome. Recently, the use of single nucleotide polymorphism (SNP) markers for linkage analysis based on next-generation sequencing (NGS) was reported (Chen et al., 2017; Chen et al., 2020; Li et al., 2020). The genes HBA1 and HBA2 of α-thalassemia are located at the end of the p arm of chromosome 16. The numbers of SNPs near the telomere in chromosome are insufficient. Therefore, in these studies using NGS, the target genes were directly detected using other methods. Neither genetic testing nor linkage analysis used alone is the best solution for PGT of α-thalassemia. Also, none of the above methods can undergo aneuploidy screening simultaneously. SNP array is a high-throughput technology that usually contains hundreds of thousands of SNPs covering the entire genome. Quantitative analysis of B allele has been successfully employed for chromosome aneuploidy screening, and analysis of whole-genome haplotype can be established by SNP typing.

In this study, we established a comprehensive PGT approach for SEA-type α-thalassemia by fluorescent Gap-PCR combined with SNP array, which can directly detect SEA-type α-thalassemia along with linkage analysis as well as aneuploidy screening.

## Materials and methods

### Patient population

This study included 25 families that underwent 26 PGT biopsy cycles from June 2021 to February 2022. In these families, the average maternal age was 30.11 ± 3.30 years (range, 23–39) and the average paternal age was 31.96 ± 3.73 years (range, 24–39). In the 25 families, both couples were carriers of SEA-type α-thalassemia. In the biopsy cycles, routine clinical service of SEA-type α-thalassemia PGT was performed using the conventional fluorescent Gap-PCR method independent of this study.

The study protocol was approved by the ethics committee of the Faculty of Medical Research Service, the First Affiliated Hospital of Sun Yat-sen University, China. All couples were given genetic counseling before treatment and were informed of the treatment protocol and procedure as well as the misdiagnosis rate of PGT. All patients were informed about relevant risks and provided written informed consent.

### IVF–embryo transfer cycle, blastocyst biopsy, and whole-genome amplification

Ovarian stimulation was performed as previously reported (Xu et al., 2009; Hou et al., 2019). Retrieved metaphase II oocytes were subjected to intracytoplasmic sperm injection. Fertilization status was observed at 16–18 h after insemination, and all 2PN embryos were submitted for blastocyst culture. About 6–10 trophectoderm cells were biopsied in blastocyst stage. All blastocysts were vitrified following biopsy, whereas the cells for biopsy were placed in 3.5 μL of phosphate-buffered saline. A RELI-g Single Cell Kit (QIAGEN, Hilden, Germany) was used for whole-genome amplification (WGA).

### Fluorescent Gap-PCR analysis for SEA-type α-thalassemia

WGA products were either used for fluorescent PCR identification immediately or stored at −20°C. Amplification involved the use of three α-thalassemia SEA primers. The S1 and S3 primers flank the SEA deletion, whereas the S2 primer anneals within the deleted area. The specific process and results of primer sequences and Gap-PCR are the same as those of our group’s earlier published studies (Deng et al., 2006; Xu et al., 2009) (Table 1).

**TABLE 1:** Primer Sequence Used (5′-3′) and PCR procedures in Gap-PCR for SEA-type α-thalassemia.

Primer sequence	PCR cycle	Product size
S1: gtg​ttc​tca​gta​ttg​gag​gga​a	2 min 96°C, 10 × (45 s 96°C, 45 s 60°C, 60 s 72°C), 25 × (45 s 94°C, 45 s 60°C, 60 s 72°C), 72°C 7 min	
S2: 5′-FAM-gacacgcttccaatacgctta		280bp
S3: 5′-HEX-ctactgcagccttgaactcc		178bp

PCR, polymerase chain reaction.

### Peripheral blood DNA extraction, SNP array analysis, and aneuploidy screening

Peripheral blood (2 mL) was collected from each couple and placed in an EDTA anticoagulant tube for genomic DNA preparation. Genomic DNA was extracted using the T Guide Large Volume Blood Genomic DNA Kit (TIANGEN, Beijing, China).

The peripheral blood DNA and WGA products after two rounds of amplification were treated according to the manufacturer’s instructions (Infinium^®^ HD Assay Super, Manual Protocol, Illumina, San Diego, CA, United States). After termination with a series of enzymes, denatured DNA and the peripheral blood DNA obtained from the parents were hybridized with the HumanCytoSNP-12 BeadChip (Illumina, United States), which contains approximately 300,000 SNPs with an average distance of 9.7 kb. Subsequently, the bead chips were scanned using an Illumina iScan Bead Array Reader (Illumina, United States). The scanning results were processed using the B-allele frequency and the log R ratio using Illumina GenomeStudio (Illumina, United States) to analyze the copy number of the chromosomes.

### Haplotype analysis of HBA1 + HBA2 genes

**FIGURE 1:**
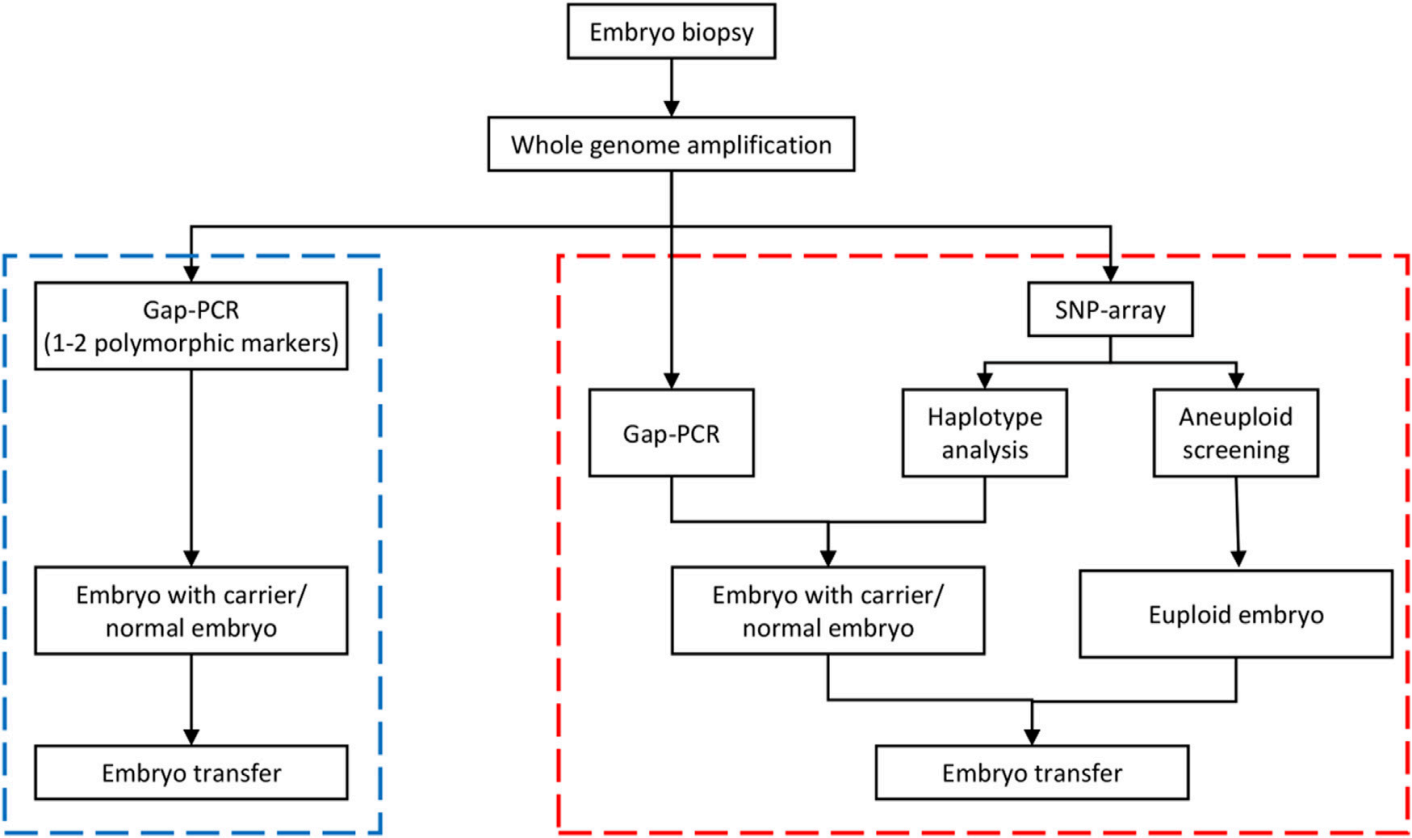
Workflow of the comprehensive preimplantation genetic testing (PGT) approach for SEA-tvpe a-thalassemia. The blue dotted box on the left is the Gap-polymerase chain reaction (PCR)-hased method flow, whereas the red dotted box if the nght is the comprehensive detection scheme. In the comprehensive detection scheme, the PCR detection step is the same as the conventional PCR detection. The comprehensive approach actually combined haplotype analysis and aneuploidy screening on the basis of PCR detection. If the Gap-PCR result was not consistent with the haplotype analysis result in an embryo sample, the biopsy sample was retested or the embryo was rebiopsied before testing. SNP, single nucleotide polymorphism.

**FIGURE 2:**
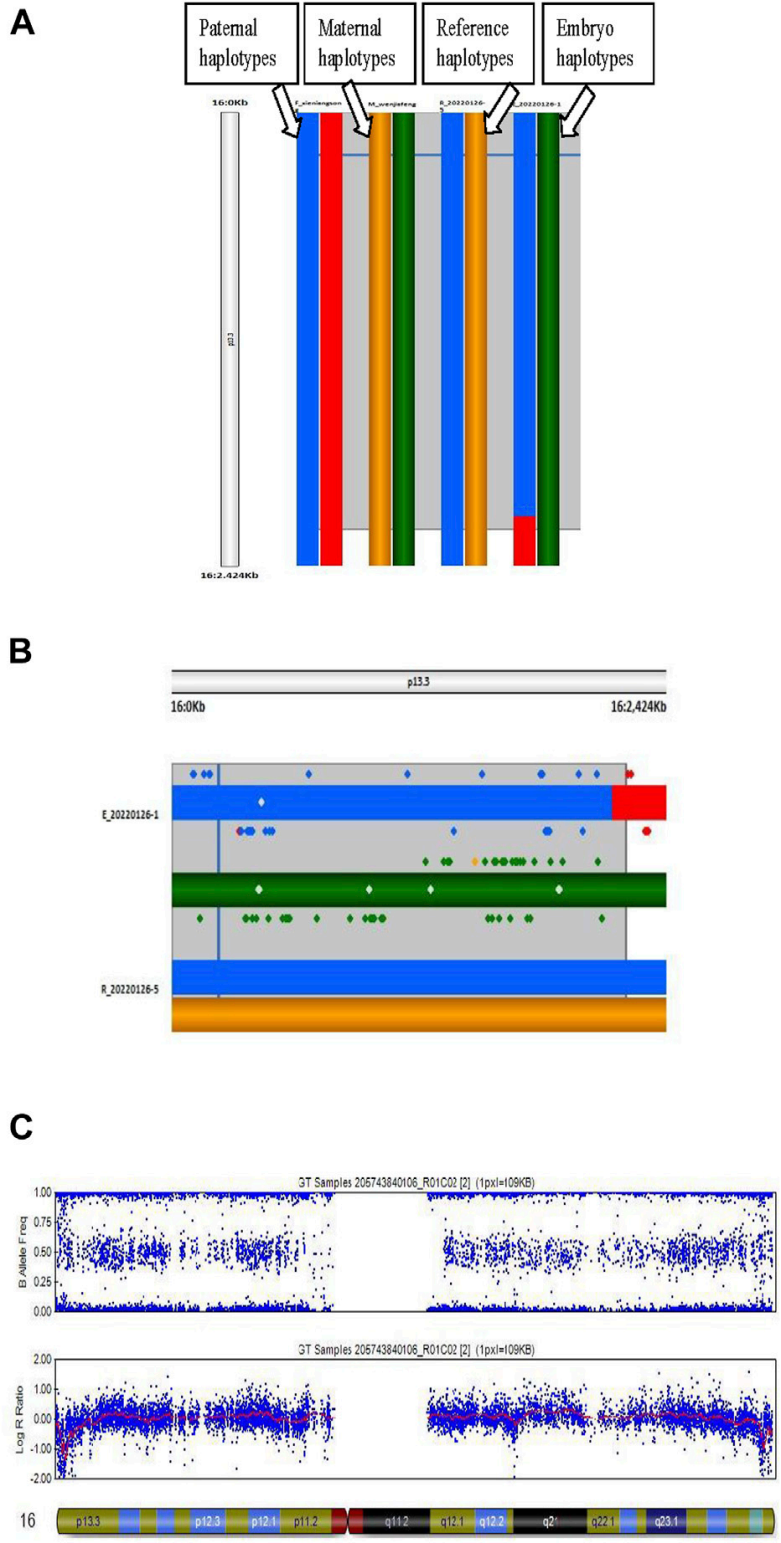
Haplotype analysis and copy number variation (CNV) in an embryo. **(A)** Process of haplotype analysis. Reference (R_20220126–5) is the haplotype of a homozygous affected embryo, the blue band is the haplotype from the father, and the yellow band is the haplotype from the mother. Thus, the blue and yellow bands are the risk haplotypes from the parents. **(B)** Details of the haplotype of the reference and embryo sample. E_20220126–1 is a heterozygous carrier due to the paternal risk haplotype. **(C)** Results of CNV of chromosome 16 in an embryo.

The genotypes of all SNP gene loci in the couples and their embryos were acquired using GenomeStudio (Illumina, United States). Informative SNPs within a 2-Mbp region flanking the HBA1 + HBA2 genes were used to build the family haplotypes. Informative SNP was defined as a heterozygous SNP genotype in one parent (such as AB) while being homozygous in the other (such as AA/BB). Embryos with gap-PCR results showing (−/− or αα/αα) were preferentially used as reference samples and assumed to have no recombination for phasing purpose. The rest of the embryos were compared with haplotype to determine whether they carried the risk haplotype. Figure 1 presents the workflow of the comprehensive method. Figure 2 shows the haplotype analysis and copy number variation results in embryos.

## Results

### Clinical characteristics of PGT cycles

A total of 26 biopsy cycles were performed. The average number of biopsied embryos was 6.62 ± 3.00. The clinical pregnancy rate of each frozen embryo transfer (FET) was 57.7% (15/26). One case had an early miscarriage, and the abortion product was not tested in any way. The number of healthy babies born was nine, and the rest were still in pregnancy. Amniotic fluid was extracted during gestational weeks 18–22 for prenatal diagnosis in six families; however, the rest of the families refused to the extraction. The results of prenatal diagnosis confirmed the PGT results. Details of the results are presented in Table 2.

**TABLE 2:** Overall clinical characteristics of preimplanation genetic testing (PGT) cycles.

Clinical characteristic	
No. of patients/cycles	25/26
Maternal age (years, mean ± SD, min–max)	30.11 ± 3.30 (23–39)
Paternal age (years, mean ± SD, min–max)	31.96 ± 3.73 (24–39)
No. of oocyte retrieved	17.50 ± 10.05
No. of biopsy	6.62 ± 3.00
Clinical pregnancy rate per transfer (%)	57.7 (15/26)
Spontaneous miscarriage rate (%)	6.67 (1/15)
Biochemical pregnancy rate (%)	6.67 (1/15)
No. of babies born	9

### Results of fluorescent Gap-PCR, haplotype analysis for SEA-type α-thalassemia, and aneuploidy screening

The homozygous normal and homozygous affected rates detected by Gap-PCR were both 22.7% (39/172), and the rate of heterozygous carriers was 54.7% (94/172). In haplotype analysis, the homozygous normal, heterozygous carrier, and homozygous affected rates were 22.7% (39/172), 54.1% (93/172), and 23.3% (40/172), respectively. The consistency rate of the two test results was 94.2% (162/172). Among the 10 embryo samples with discordant results, 7 were found to be aneuploidy on chromosome 16 via whole-chromosome haplotype analysis and aneuploidy screening. The remaining three samples were retested by PCR. Among them, two exhibited a single peak of 280 bp (αα/αα) and 178 bp (−/−) in the first PCR, respectively, but both results showed two peaks of 178 and 280 bp (--/αα) in the second PCR. Both the results of the second PCR matched the haplotype analysis results. For these two samples, by comparing the results of haplotype analysis with the two PCR results, ADO was observed. In the first PCR, one of these two samples was diagnosed as a homozygous normal embryo, whereas the other was diagnosed as a homozygous affected embryo. However, the results of both the haplotype analysis and second PCR proved that the two samples were heterozygous carriers. Thus, the Gap-PCR ADO rate was estimated to be 1.16% (2/172).

For the last embryo sample, the second PCR result still exhibited a single peak of 178 bp (−/−). Furthermore, the call rate of this sample was 0.88 after SNP array detection, suggesting that the WGA amplification quality was poor. Therefore, a second biopsy sample was taken for the embryo. The PCR result of the rebiopsied sample was consistent with that of the previous one. The result of the haplotype analysis was consistent with those of the three PCRs when the call rate was 0.95. Table 3 presents the results in detail. For aneuploidy screening, of the 172 embryos, 170 were successfully detected, with 105 embryos being euploid and the euploid rate being 61.8% (105/170).

**TABLE 3:** Fluorescent Gap-PCR and haplotype analysis for SEA-type α-thalassemia in the 10 embryo samples with discordant results.

Embryo No.	Gap-PCR	Haplotype	Recheck the results	Final results	Analysis of cause
1	280bp^*^ (αα/αα) ^#^	maternal heterozygous	PCR was performed on the amplified sample again and the result showed two peaks of 178bp280bp.	--/αα	ADO of the mutant allele
2	178bp280bp (--/αα)	abnormal homozygous	The CNV and haplotype of chromosome 16 were reviewed. Chromosome 16 CNV was arr 16p13.3q23.1 (1-79,652,129) × 3 mat. According to the haplotype, the error occurred in the first meiosis (MI).	--/--/αα	BPH trisomy leaded to confusing haplotype in chromosome 16.
3	178bp280bp (--/αα)	heterozygous	The CNV and haplotype of chromosome 16 were reviewed. Chromosome 16 CNV was arr(16) × 3 mat. According to the haplotype, the error occurred in the first meiosis (MI).	--/--/αα	BPH trisomy leaded to confusing haplotype in chromosome 16.
4	178bp280bp (--/αα)	abnormal homozygous	The CNV and haplotype of chromosome 16 were reviewed. Chromosome 16 CNV was arr(16) × 3 mat. According to the haplotype, the error occurred in the first meiosis (MI).	--/--/αα	BPH trisomy leaded to confusing haplotype in chromosome 16.
5	178bp280bp (--/αα)	abnormal homozygous	The CNV and haplotype of chromosome 16 were reviewed. Chromosome 16 CNV was arr(16) × 3 mat. According to the haplotype, the error occurred in the first meiosis (MI).	--/--/αα	BPH trisomy leaded to confusing haplotype in chromosome 16.
6	178bp (--/--)	maternal heterozygous	PCR was performed on the amplified samples again and the results showed two peaks of 178bp280bp.	--/αα	ADO of the normal allele
7	178bp280bp (--/αα)	heterozygous	The CNV and haplotype of chromosome 16 were reviewed. Chromosome 16 CNV was arr(16) × 3 mat. According to the haplotype, the error occurred in the first meiosis (MI).	--/--/αα	BPH trisomy leaded to confusing haplotype in chromosome 16.
8	178bp (--/--)	maternal heterozygous	PCR was performed on the amplified sample again and the result showed only one peak of 178bp. The embryo was biopsied again. PCR was performed on the amplified sample and the result still showed only one peak of 178bp. Haplotype was performed on the re-biopsy sample. The result was consistent with the three PCRs.	--/--	The call rate was 0.88, poor quality of WGA amplification caused SNP typing errors.
9	178bp280bp (--/αα)	normal homozygous	The CNV and haplotype of chromosome 16 were reviewed. Chromosome 16 CNV was arr(16) × 3 mat. According to the haplotype, the error occurred in the first meiosis (MI).	--/--/αα	BPH trisomy leaded to confusing haplotype in chromosome 16.
10	280bp (αα/αα)	heterozygous	The CNV and haplotype of chromosome 16 were reviewed. Chromosome 16 CNV was arr(16) × 1 mat.	αα	The maternal source of chromosome 16 was missing. Only the non-risk haplotype of paternal source was remained. So, PCR can only amplify a 280bp fragment.

^*^: target fragment peak, ^#^: thalassaemia genotype, ADO, allele drop-out; BPH, both parental homologs; WGA, whole genome amplification; PCR, polymerase chain reaction; CNV, copy number variation; SNP, single nucleotide polymorphic.

### Number of informative SNPs of Cyto-12 array in sample population

The HBA genes are located at 16p13.3 (GRCh37 16:222846–227521) at the end of the p arm of chromosome 16. The Cyto-12 array has 407 customized SNPs in the 2-Mbp region flanking the HBA1 + HBA2 genes, including 10 in the genes, 42 in the upstream 5′ region, and 355 in the downstream 3′ region. According to the haplotype statistics of the 25 families, no informative SNPs were observed within the HBA genes. In the downstream 3′ 2Mb region of the genes, the average numbers of maternal and paternal informative SNPs were 42.52 ± 14.561 and 34.96 ± 10.907, respectively. Within the upstream 5′ 2Mb region of the genes, the average numbers of maternal and paternal informative SNPs were 2.36 ± 2.177 and 2.88 ± 2.00, respectively. Table 4 presents the results in detail.

**TABLE 4:** Number of informative SNPs (the HBA genes and 2-Mbp region flanking the gene) of Cyto-12 array in detected families.

Family	Left flanking 5′	HBA1+HBA2	Left flanking 3′	Left flanking 5′	HBA1+HBA2	Left flanking 3′
	Maternal informative SNPs			Paternal informative SNPs		
1	0	0	39	0	0	25
2	1	0	44	0	0	33
3	0	0	47	0	0	24
4	1	0	27	4	0	60
5	1	0	36	5	0	27
6	4	0	59	5	0	25
7	1	0	64	5	0	34
8	0	0	43	0	0	44
9	3	0	37	5	0	25
10	3	0	33	3	0	31
11	2	0	49	7	0	41
12	6	0	46	2	0	21
13	6	0	37	1	0	33
14	2	0	11	2	0	46
15	3	0	16	2	0	44
16	1	0	51	1	0	20
17	1	0	45	4	0	33
18	1	0	49	4	0	33
19	1	0	50	8	0	48
20	1	0	68	5	0	24
21	8	0	42	1	0	31
22	4	0	67	1	0	26
23	1	0	21	1	0	52
24	6	0	30	2	0	45
25	2	0	52	4	0	49

SNP, single nucleotide polymorphic.

## Discussions

In this study, we presented a comprehensive PGT approach for SEA-type α-thalassemia by fluorescent Gap-PCR combined with SNP array, which can directly detect SEA-type α-thalassemia along with linkage analysis as well as aneuploidy screening.

The detection methods of SEA-type α-thalassemia PGT evolved from early PCR technologies (Deng et al., 2006; Wirawit et al., 2012; Ta-Hsien et al., 2017), to STR polymorphic markers (Aspasia et al., 2012), then to recent SNP haplotype analysis based on NGS (Chen et al., 2017; Chen et al., 2020; Li et al., 2020). Direct detection of target genes potentially leads to misdiagnosis due to ADO. Haplotype analysis based on polymorphic markers can reduce the risk of misdiagnosis caused by ADO. However, the distribution and number of STRs are very limited, and high-throughput detection cannot be achieved. Furthermore, SNP haplotype analysis based on NGS is only limited to haplotype construction in the HBA genes and 1–2-Mbp region flanking the regions of the genes. More importantly, none of the aforementioned methods can undergo aneuploidy screening simultaneously.

In the present study, PGT-M was performed on SEA-type α-thalassemia. On the basis of fluorescent Gap-PCR, haplotype analysis and aneuploidy screening were additionally performed. In particular, PCR was used to directly detect the target genes (HBA1 + HBA2), and 300,000 SNPs customized by SNP array were used to perform chromosome-wide haplotype analysis and aneuploidy screening of embryos. Gap-PCR is a reliable method for prenatal diagnosis of α-thalassemia (Ko et al., 1992). Since 2006, our center has been adopting fluorescent Gap-PCR for SEA-type α-thalassemia PGT (Deng et al., 2006), which is easy to operate and time-saving. By using fluorescent-labeled primers and a genetic analyzer to analyze the amplified PCR products, the detection sensitivity of a few PCR products has improved (Xu et al., 2009). The mature blastocyst biopsy technique can provide more trophectoderm cells for detection, which significantly reduces the risk of misdiagnosis and missing heterozygotes caused by ADO. ADO is an inherent problem of PCR that cannot be completely eliminated. In this study, there were still two embryos with wrong fluorescent Gap-PCR results due to ADO. SNP array can be employed to construct genome-wide haplotypes and conduct linkage analysis in the target gene and any size region flanking the gene, which can effectively reduce the impact of ADO on the direct detection of the variation site. However, a proband is required to construct the haplotype of the family. Fetuses with homozygous deletion cannot survive, and offspring of heterozygous carriers cannot be probands as the risk haplotype cannot be determined. Therefore, either homozygous normal offspring are required or samples from the parents of the previous generation of both the husband and wife need to be traced (if both parents of the husband and wife are heterozygous carriers, the risk chromosome cannot be judged effectively). This would be an inefficient and bulky project. Therefore, determining how to construct the haplotype of the carrier family in the most simplified manner is the key to conduct the haplotype analysis in SEA-type α-thalassemia PGT. Our scheme was to choose homozygous affected or homozygous normal (wild-type) embryos as probands according to the results of fluorescent Gap-PCR. If neither of the aforementioned two conditions existed, the two haplotypes of heterozygous carrier embryos are compared with each other. It is necessary to determine whether they meet the inference that one haplotype is the risk haplotype of the father/mother and the other is the non-risk haplotype of the mother/father. However, special care needs to be taken as heterozygous variants may appear as homozygous normal or homozygous affected false results due to ADO of Gap-PCR. ADO misdiagnosis can also be corrected by haplotyping sibling embryos. Therefore, at least two or more embryos are needed during the PGT-M cycle. Furthermore, haplotype analysis can prompt exogenous DNA contamination, which is also a potential risk factor for misdiagnosis caused by PCR.

The genes HBA1 and HBA2 of α-thalassemia are located at 16p13.3, close to the end of the p arm of chromosome 16, and less than 0.3 Mb from the upstream 5′ region to the end of the p arm of the genes. The array customized 36 SNPs in the upstream region; therefore, informative SNPs that can be used to construct haplotypes were limited. In our sample population, 2.36 ± 2.177 maternal informative SNPs and 2.88 ± 2.00 paternal informative SNPs were detected, respectively. Because there are fewer SNPs at the upstream 5′ region of the HBA genes, for haplotype analysis, gene recombination is a risk factor for analysis errors. Bjarni et al. reported that there were 173 and 38 paternal and maternal recombination hotspots, respectively, within 2 Mb upstream and downstream of the HBA1 + HBA2 genes (CRGh37 16:0–2227521), but they all occurred in the near-centromeric region downstream of the HBA genes.al. They used samples from the Icelandic population (Bjarni et al., 2019). We also conducted statistical analysis on the data of embryo samples of the IVF-PGT population in our center, showing that 35 were paternal and 2 were maternal hotspots, both of which were located downstream of the HBA genes (Ma et al., 2023). It can be deduced that the risk of gene recombination near the telomeric region upstream of HBAs is very small. In our data, no upstream HBA recombination was observed. However, there were three couples with no parental informative SNPs at the 5′ end, and one couple had no paternal informative SNPs. Due to the risk of recombination and ADO, the risk of misdiagnosis was increased in the four families. Thus, for such families, we prioritized the selection of homozygous normal embryos for transplantation to minimize the adverse effects of misdiagnosis. In addition, due to few polymorphic markers near the telomeric region, the poor quality of sample WGA amplification may affect the accuracy of SNP typing, resulting in errors in haplotype analysis. Our pedigree sample size was too small and needs to continue to be evaluated on larger samples basis for recombination near the telomeres of HBA genes and its impact on haplotype analysis. Therefore, combined with the special situation of the HBA genes (influence of the chromosomal position of the genes on haplotype analysis), we suggest that it is necessary to combine Gap-PCR with haplotype analysis for SEA-type α-thalassemia PGT.

The advantages of our comprehensive PGT approach were as follows: additional experimental operations are not needed, and aneuploidy can be screened only via quantitative analysis of the customized 300,000 SNPs distributed on 23 chromosomes. At present, uncertainties still exist regarding the efficacy of PGT-A routinely applied in IVF population (Alexander et al., 2018; Shelby et al., 2018; Miriam et al., 2020; Yan et al., 2021). In 2019, our group reported that aneuploidy screening in PGT for monogenic diseases among women below 35 years old significantly increased persistent pregnancy/live birth rate in their first FET cycles, reducing the time interval from the first ET to pregnancy, compared with PGT-M without aneuploidy screening (Hou et al., 2019). Furthermore, genome-wide haplotype analysis can be employed to characterize extensive chromosomal aberrations in the whole genome as well as the parental origin and occurrence period of various chromosomal errors. This is not possible with PGT-A, in which only copy number analysis is conducted (David et al., 2019; Lin et al., 2021; Olga et al., 2021). In addition, chromosomal ploidy errors can interfere with our judgment of target gene variations. It is well known that aneuploidy on chromosome 16 is a common chromosomal abnormality in prenatal period. HBA genes are located on chromosome 16; therefore, chromosomal ploidy error may interfere with the detection results of α-thalassemia. In this study, the PCR results of 7 embryos were inconsistent with the haplotype results as chromosome 16 was aneuploidy. The genome-wide haplotype analysis revealed that the extra chromosome 16 of the six embryos came from their mothers, which occurred in meiosis. It can be determined the haplotypes in the region near the HBA genes formed three distinct parental haplotypes [i.e., both parental homologs (BPH)] (David et al., 2019; Daniel et al., 2021) because of chromosomal duplication caused by meiosis errors, leading to inconsistency between the two results. In addition, the embryo with monosomy 16 lost the chromosome of its maternal origin.

The biggest difference between haplotype analysis based on SNP array and NGS-based SNP haplotyping is that the former is a genome-wide haplotype analysis whereas the latter only includes haplotypes in a region or a gene and its near region (usually 1–2 Mbp). Because SNP array constructs the haplotype of the whole genome, the range of haplotype analysis in the target region can be changed if necessary. Genome-wide haplotype analysis can also illustrate the whole picture of chromosomes. In recent years, a general method based on NGS, such as OnePGT, has also emerged, which can automatically complete haplotyping and copy number analysis of the whole genome (Heleen et al., 2019; L; De Witte et al., 2022; Heleen et al., 2022). The implementation of this technology still requires performance evaluation through prospective trials.

In conclusion, Gap-PCR combined with haplotype analysis based on SNP array for SEA-type α-thalassemia is feasible and necessary. This approach combines direct detection of target genes and haplotype analysis to minimize the risk of misdiagnosis caused by ADO and gene recombination. No additional experimental operation was required for aneuploidy screening. However, a larger sample size is needed to examine the impact of recombination near the telomeric region upstream of the HBA genes on the detection results.

## Data Availability

The original contributions presented in the study are publicly available. This data can be found here: https://ngdc.cncb.ac.cn/search/?dbId=omix&amp;q=OMIX004628&amp;page=1.
