# Supplementary material for: A comprehensive preimplantation genetic testing approach for SEA-type α-thalassemia by fluorescent gap-polymerase chain reaction combined with haplotype analysis
Source: Front Genet. 2023 Nov 23;14:1248358. doi: 10.3389/fgene.2023.1248358 (PMC10702134; doi:10.3389/fgene.2023.1248358)
Supplement: Supplementary file 1 [file Table1.pdf]

Table S1. Fluorescent Gap-PCR and haplotype analysis for SEA-type α-thalassemia in the 10 embryo samples with discordant results

Embryo No.	Gap-PCR	Haplotype	Recheck the results	Final results	Analysis of cause
1	280bp* (αα/αα) #	maternal heterozygous	PCR was performed on the amplified sample again and the result showed two peaks of 178bp280bp.	--/αα	ADO of the mutant allele
2	178bp280bp (--/αα)	abnormal homozygous	The CNV and haplotype of chromosome 16 were reviewed. Chromosome 16 CNV was arr 16p13.3q23.1(1-79,652,129)×3 mat. According to the haplotype, the error occurred in the first meiosis(MI).	--/--/αα	BPH trisomy leaded to confusing haplotype in chromosome 16.
3	178bp280bp (--/αα)	heterozygous	The CNV and haplotype of chromosome 16 were reviewed. Chromosome 16 CNV was arr(16)×3 mat. According to the haplotype, the error occurred in the first meiosis(MI).	--/--/αα	BPH trisomy leaded to confusing haplotype in chromosome 16.
4	178bp280bp (--/αα)	abnormal homozygous	The CNV and haplotype of chromosome 16 were reviewed. Chromosome 16 CNV was arr(16)×3 mat. According to the haplotype, the error occurred in the first meiosis(MI).	--/--/αα	BPH trisomy leaded to confusing haplotype in chromosome 16.
5	178bp280bp (--/αα)	abnormal homozygous	The CNV and haplotype of chromosome 16 were reviewed. Chromosome 16 CNV was arr(16)×3 mat. According to the haplotype, the error occurred in the first meiosis(MI).	--/--/αα	BPH trisomy leaded to confusing haplotype in chromosome 16.
6	178bp (--/--)	maternal heterozygous	PCR was performed on the amplified samples again and the results showed two peaks of 178bp280bp.	--/αα	ADO of the normal allele

7	178bp280bp (--/αα)	heterozygous	The CNV and haplotype of chromosome 16 were reviewed. Chromosome 16 CNV was arr(16)×3 mat. According to the haplotype, the error occurred in the first meiosis(MI).	--/--/αα	BPH trisomy leaded to confusing haplotype in chromosome 16.
8	178bp (--/--)	maternal heterozygous	PCR was performed on the amplified sample again and the result showed only one peak of 178bp.The embryo was biopsied again. PCR was performed on the amplified sample and the result still showed only one peak of 178bp. Haplotype was performed on the re-biopsy sample. The result was consistent with the three PCRs.	−−/−−	The call rate was 0.88, poor quality of WGA amplification caused SNP typing errors.
9	178bp280bp (--/αα)	normal homozygous	The CNV and haplotype of chromosome 16 were reviewed. Chromosome 16 CNV was arr(16)×3 mat. According to the haplotype, the error occurred in the first meiosis(MI).	--/--/αα	BPH trisomy leaded to confusing haplotype in chromosome 16.
10	280bp (αα/αα)	heterozygous	The CNV and haplotype of chromosome 16 were reviewed. Chromosome 16 CNV was arr(16)×1 mat.	αα	The maternal source of chromosome 16 was missing. Only the non-risk haplotype of paternal source was remained. So, PCR can only amplify a 280bp fragment.

*:target fragment peak, #:thalassaemia genotype, ADO: allele drop-out, BPH: both parental homologs, WGA: whole genome amplification, PCR: polymerase chain reaction, CNV: copy number variation, SNP: single nucleotide polymorphic.
